# Myo1e/f regulate phagocytic podosomes to promote efficient cup closure in macrophages

**DOI:** 10.64898/2026.04.30.721640

**Published:** 2026-05-01

**Authors:** TC Paul, YM Loyd, SE Chase, TW O’Connor, CM Hobson, RM Lee, D Vorselen, M Krendel

**Affiliations:** 1 Cell and Developmental Biology Department, State University of New York Upstate Medical University, Syracuse, NY, USA; 2 Advanced Imaging Center at Janelia Research Campus, Howard Hughes Medical Institute, Ashburn, VA, USA; 3 Department of Cell Biology and Immunology, Wageningen University, Netherlands

## Abstract

Phagocytosis requires coordinated remodeling of the actin cytoskeleton to generate protrusive and contractile forces that drive target engulfment. Class I myosins Myo1e and Myo1f (Myo1e/f) have been implicated in linking the plasma membrane to the actin network, but their specific roles during Fc-receptor-mediated phagocytosis remain unclear. Using CRISPR-edited RAW 264.7 macrophages lacking Myo1e and Myo1f, we show that double knockout (dKO) cells exhibit markedly reduced uptake of IgG-coated beads, a phenotype that is partially rescued by re-expression of either myosin. Lattice-light-sheet and confocal imaging revealed distinct F-actin architectures corresponding to the various stages of cup progression, including basal podosome-like adhesions, individual phagocytic podosomes (actin teeth) along the rim of the cup, and a contractile phagocytic ring formed by the reorganization of podosomes into a higher-order network. In Myo1e/f-deficient cells, podosome formation was diminished, actin teeth were largely absent, and the phagocytic ring formed prematurely, which was often accompanied by stalled cup progression and repeated engulfment attempts. Myo1e/f localized both to podosomes and to the inner surface of the phagocytic ring, non-muscle myosin II (NM2) localized to the outer surface, and the absence of Myo1e/f correlated with the diffuse distribution of NM2. In addition, Myo1e/f-deficient macrophages exhibited increased trogocytosis of antibody-opsonized HL-60 cells, indicating a shift from whole-target engulfment toward partial target ingestion. These results suggest that Myo1e/f coordinate spatial and temporal transitions between protrusive and contractile actin networks, thereby ensuring efficient phagocytic cup progression. Our findings highlight a dual role for Myo1e/f in adhesion regulation and force balance during macrophage phagocytosis.

## Introduction

Phagocytosis is a complex and dynamic process in which specialized immune cells, termed professional phagocytes, engulf pathogens, apoptotic cells, and cellular debris^1–3^. This process is initiated when receptors on the phagocyte bind to specific ligands on potential phagocytic targets, including opsonins (surface-marking proteins) such as complement proteins or antibodies^4^.

In Fc-receptor (FcR) mediated phagocytosis, the Fc portion of an antibody bound to a target engages FcRs on the phagocyte surface^5^. Following this initial contact, FcRs cluster to form adhesions with the target^6^. Subsequently, the cell membrane extends around the target, forming the actin-rich phagocytic cup, which expands until the target is fully internalized into a phagosome^4^. The contents of the phagosome are then bound for degradation through fusion with lysosomes^7^.

Recent studies have identified podosome-like structures (dynamic actin-based adhesions) during the formation and progression of the phagocytic cup^8–10^. Podosomes consist of a core composed of branched actin filaments (F-actin) surrounded by a ring composed of adhesion proteins such as paxillin, vinculin, and talin and are involved in cell adhesion and migration^11^. They are characteristic of hematopoietic cells such as macrophages^12^ and osteoclasts^13^. Structures that are similar in size, protein composition, and protrusive activity to podosomes have been detected at multiple stages of FcR-mediated phagocytosis, from initial target adhesion^8^ to cup extension and closure ^9,10^, where they may contribute to protrusive force generation within the cup.

Class-I myosins, single-headed myosin motor proteins, are commonly found in association with branched F-actin. Using a positively charged tail homology 1 (TH1) domain, they bind to anionic phospholipids such as PI(4,5)P_2_ in the plasma membrane^14–16^. By interacting with branched actin networks via their motor domains, they generate a connection between actin filaments and the plasma membrane. Two members of the class I myosin family, myosin 1e and myosin 1f (Myo1e/f), localize to actin-rich adhesions within the phagocytic cup and coincide with podosome-like protrusive structures^8^. Previous studies have shown that Myo1e/f help maintain membrane tension^8,17^, regulate F-actin organization during phagocytosis, and promote efficient target engulfment^8,18^. Findings from our recent study using bone marrow derived macrophages (BMDM) from Myo1e/f dKO mice suggest that Myo1e/f and non-muscle myosin-II generate complementary forces (protrusive and constrictive, respectively) during phagocytosis^18^. However, since BMDMs exhibit a relatively gentle force signature and fairly uniform actin distribution, with less pronounced podosome-like structures at the rim of the cup, using these primary cells made it difficult to resolve specific myosin-driven deformation events and actin reorganization. To precisely define the spatiotemporal contributions of Myo1e/f to phagocytic cup architecture, we employed the RAW 264.7 macrophage model, which provides a highly reproducible and distinct pattern of target deformation and actin dynamics ideal for mechanical studies.

Using CRISPR/Cas9-generated Myo1e/f double-knockout RAW macrophages, live lattice light-sheet microscopy, microparticle traction force microscopy^19^, and confocal imaging of actin architecture in cells fixed after target particle engulfment, we reveal how loss of Myo1e/f alters the balance between formation of individual protrusive podosome-like structures and their assembly into contractile rings within phagocytic cups, likely leading to reduced target uptake. These findings define a mechanistic role for Myo1e/f in orchestrating the cytoskeletal transitions that enable efficient phagocytic cup progression.

## Results

### Generation and characterization of the myosin1e/f double knock-out RAW264.7 cells

**Figure 1.**
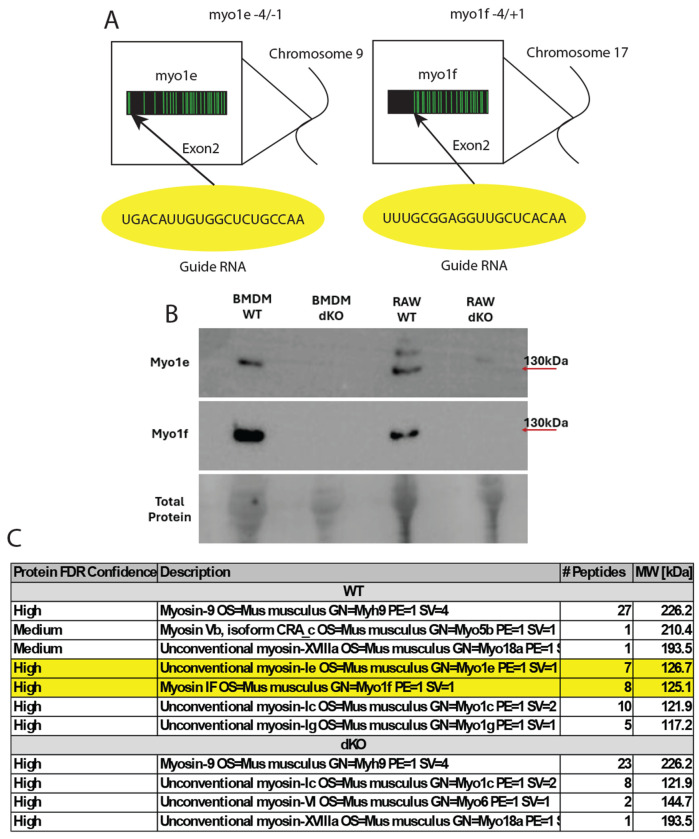
CRISPR-Cas 9 targeting of exon 2 in Myo1e and Myo1f generates myosin double knockout in RAW264.7 macrophages. **(A)** Schematic of CRISPR–Cas9 editing strategy. Guide RNAs targeting exon 2 of Myo1e (left) and Myo1f (right) (yellow) introduced small indels (‒4/‒1 for Myo1e and ‒4/+1 for Myo1f), resulting in frameshifts and premature stop codons in both genes. The indels shown correspond to the edits identified in the RAW dKO clone (B1) that was used for most experiments in this study. **(B)** Western blot validation of Myo1e/f loss. Comparison of the edited clone (B1) with WT RAW 264.7 cells and bone marrow–derived macrophages (BMDMs) confirmed loss of Myo1e and Myo1f expression. Arrows indicate the expected molecular weights of Myo1e and Myo1f. There are additional bands unique to RAW cells that are recognized by the anit-myo1e antibody. SYPRO Ruby total protein stain was used as a loading control. **(C)** Mass spectrometry confirmation of Myo1e/f knockout. SDS–PAGE gel regions corresponding to 50–200 kDa were excised and analyzed. Myo1e and Myo1f peptides were absent in dKO samples but detected in WT controls. False discovery rate (FDR) confidence, peptide counts, and predicted molecular weights are shown.

To investigate the role of Myo1e/f in phagocytosis, we generated Myo1e/f dKO RAW264.7 immortalized murine macrophages using CRISPR/Cas9 gene editing performed by Synthego. The editing introduced frameshift mutations in Exon 2 of both *Myo1e* and *Myo1f,* leading to premature stop codons (Fig.1A). Following limited-dilution cloning, two independent clones (B1 and D9) carrying stop codons in both alleles of *Myo1e* and *Myo1f* genes were identified and returned to our laboratory. The B1 clone was used as the main dKO cell line in this study.

Western blotting with antibodies against Myo1e or Myo1f confirmed the absence of Myo1e and Myo1f protein bands in dKO cells compared to wild-type (WT) controls (Fig.1B, S1B). In addition to the expected Myo1e band observed only in WT lysates, an additional band recognized by the anti-Myo1e antibody was detected in both WT and dKO cells. To determine whether this band represented Myo1e or an alternatively spliced Myo1e isoform, we excised the 50-250 kDa region from SDS-PAGE gels and performed proteomic analysis. Myo1e and Myo1f peptides were detected in WT but not dKO samples, confirming the specificity of the knockout (Fig.1C).

To further control for potential clonal variability, we performed a second round of limited-dilution cloning from the edited RAW264.7 pool. Twenty-two additional clones were isolated, and eleven were screened by Western blotting to identify additional Myo1e/f-deficient lines (Fig. S1A).

### Myo1e/f deficiency results in decreased uptake of IgG coated beads

**Figure 2.**
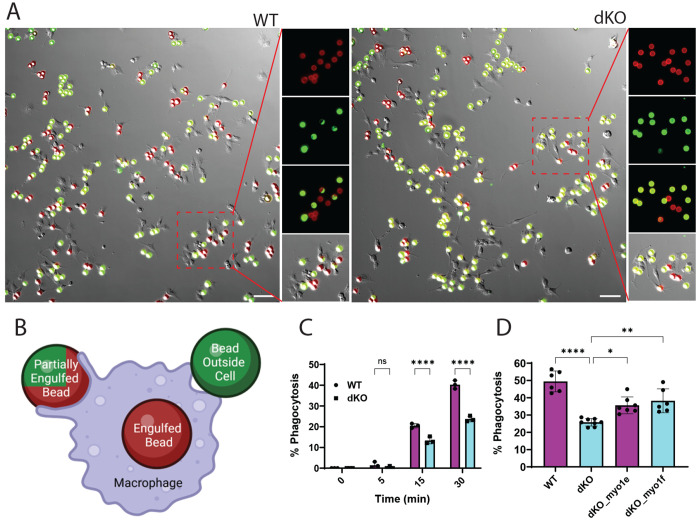
Myo1e/f double knockout impairs phagocytic efficiency in RAW 264.7 macrophages. **(A)** Representative images from the 30-min timepoint in the phagocytic efficiency assay. Merged three-channel images show an increased number of fully engulfed beads (red) in WT RAW 264.7 macrophages (left) compared to Myo1e/f dKO cells (right). A 100 × 100 μm region (red box) highlights a representative area with individual analysis channels: all beads (red), surface-exposed/unengulfed beads (green), and merged views (2-channel to highlight partial engulfment; 3-channel to show total cells vs phagocytosing cells). Scale bar: 50 μm. **(B)** Cartoon illustrating bead classification. In merged images, fully engulfed beads appear red, fully unengulfed beads appear green, and partially engulfed beads show combined red/green signal. **(C)** Time course of the phagocytic e_iciency assay. Quantification at 15 and 30 min shows a significant decrease in phagocytic efficiency in dKO cells (cyan bars, square markers) compared with WT cells (magenta bars, circle markers). Each data point corresponds to summative % of phagocytosis for an individual experiment, boxes indicate mean value, error bars – SD. N=3 experiments. **(D)** Rescue of phagocytic efficiency by reintroduction of Myo1e or Myo1f. At ~35% transfection efficiency, expression of either Myo1e or Myo1f in dKO macrophages partially restores phagocytic efficiency at the 30 min timepoint toward WT levels. Two independent experiments (N = 2) were performed, each with four wells (n = 4 per experiment). Phagocytic efficiency was quantified for each well and used for statistical analysis. Wells with transfection efficiency <10% were excluded, boxes indicate mean value, error bars – SD.). Statistical significance: ns = p > 0.05, * p < 0.05, ** p < 0.01, *** p < 0.001, **** p < 0.0001. Scale bar: 50 μm.

To determine how Myo1e/f loss affects functional phagocytosis, we performed a phagocytic efficiency assay using WT and Myo1e/f dKO (clone B1) RAW264.7 cells. Cells were incubated with 9 μm IgG-coated polystyrene beads that were centrifuged onto the cell surface to synchronize uptake (time 0). Phagocytosis was stopped by fixation at specified time points, and the exposed surface of the beads was detected by staining with fluorescent secondary antibodies to determine what fraction of all beads was fully engulfed at each time point (Fig. 2). A pronounced reduction in phagocytic efficiency was observed at the 15 and 30-minute timepoints in the Myo1e/f dKO cells compared to WT controls (Fig. 2C). To confirm that the phagocytic defect was not limited to a single dKO clone, we performed the same assay with several additional Myo1e/f-deficient clones generated from the edited cell pool. Each of these clones exhibited some degree of the phagocytosis defect (Fig.S1C-D).

To test whether the reintroduction of Myo1e or Myo1f could rescue the phagocytic defects observed in dKO cells, we transfected dKO cells with Halo-tagged Myo1e or Myo1f constructs and repeated the phagocytic efficiency assay at the 30-minute timepoint. As expected (due to an average transfection efficiency of ~35% for both constructs), re-expression of either protein only partially restored phagocytic efficiency, with Myo1f expression resulting in a slightly more pronounced rescue (Fig. 2D).

### Dynamic F-Actin Transitions During Phagocytosis

**Figure 3.**
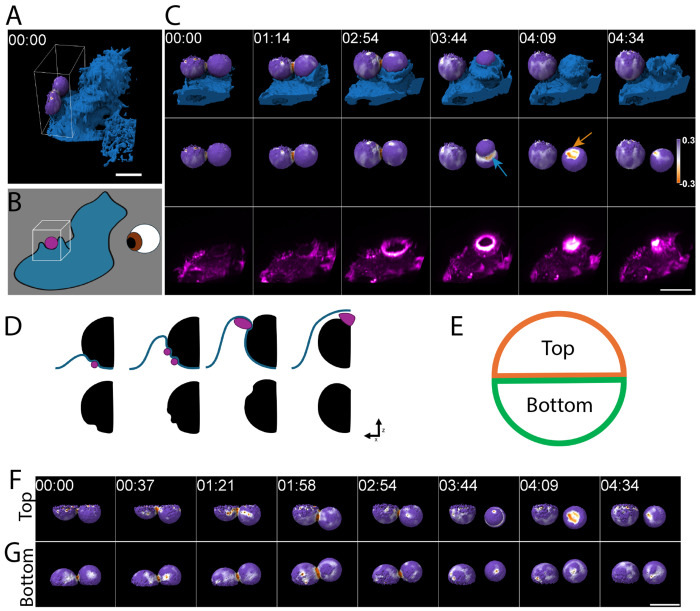
Typical progression of F-actin reorganization and bead deformation during WT phagocytosis. **(A-C)** Live-cell LLSM imaging of IgG-coated DAAMP phagocytosis in RAW 264.7 WT macrophages. **(A)** Overview of macrophages expressing mEmerald–Lifeact (blue isosurface) interacting with deformable IgG-coated DAAMPs, represented as an isosurface with a diverging purple–orange colormap representing local curvature measurements between -0.3 and 0.3 steradians. **(B)** Wireframe indicates the region of interest (ROI) corresponding to the montage shown, viewed from the angle indicated in the schematic. **(C)** The montage depicts the right bead undergoing complete engulfment and the left bead remaining at the early attachment stage. Localized bead deformations coincide spatially with F-actin hotspots, visualized using a volumetric maximum intensity projection (MIP) paletted as magenta-white heatmap to highlight areas of actin enrichment. The blue arrow highlights a phagocytic ring, and the orange arrow highlights a cap-like actin spot that forms upon cup closure. **(D)** Schematic illustrating bead (black) deformations driven by Factin-rich podosome-like structures (purple) and macrophage membrane (blue) dynamics during antibody mediated phagocytosis. **(E)** Schematic of the two viewing orientations (camera perspectives) of the DAAMPs shown in subsequent panels. **(F, G)** Volumetric rendering of the DAAMP particle as an isosurface, undergoing phagocytosis by RAW 264.7 macrophages expressing mEmerald–Lifeact represented as a MIP. Two viewing orientations were used to reconstruct the images, with the camera views pointed at the top **(F)** or bottom **(G)** hemispheres of the DAAMP undergoing phagocytosis. The purple–orange colormap denotes triangulated surface curvature between ‒0.3 and 0.3 steradians, with purple representing convex regions of the bead and orange indicating indented regions of the bead. Scale bars, 10 μm.

We next examined actin reorganization during phagocytic cup progression and the corresponding changes in the forces applied to phagocytic targets using two complementary approaches: live-cell imaging and imaging of fixed phagocytic cups. First, we used lattice light-sheet microscopy (LLSM) to observe phagocytosis in live macrophages. Fluorescently labeled deformable acrylamide-co-acrylic acid microparticles (DAAMPs) served as phagocytic targets, allowing simultaneous observation of actin dynamics and target deformation by LLSM^19,20^. Using LLSM, we imaged WT RAW264.7 macrophages expressing mEmerald-Lifeact to label F-actin as they engulfed Alexa-647–cadaverine labeled DAAMPs opsonized with IgG (Fig.3, Fig.S2, Supp Videos 1,3). In the initial phase of phagocytosis, in WT cells viewed with LLSM, F-actin-rich hotspots resembling podosomes appeared at sites of cell–bead contact and coincided with regions of deformation at the base of the bead (Fig.3F,G, Fig.S2B,C, white arrows). We refer to these early structures as basal podosomes. As the cup advanced over the bead, additional F-actin hotspots appeared near the leading edge. These structures varied in the extent of bead deformation but consistently coincided with localized indentations. At later stages engulfment, a dense band of F-actin, referred to here as the phagocytic ring, formed at the membrane’s leading edge and coincided with intense deformation of the bead surface (Fig.3C, Fig.S2B,C, blue arrows). The phagocytic ring persisted through target enclosure, after which F-actin reorganized into a cap-like structure over the top of the bead (Fig.3C, Fig.S2B,C, orange arrows), producing marked deformations likely associated with pushing the newly internalized phagosome toward the cell interior. This bright actin cap is reminiscent of the burst of actin assembly previously observed following phagosome closure in complement receptor-mediated phagocytosis, which was also proposed to propel the phagosome inward^21^.

**Figure 4.**
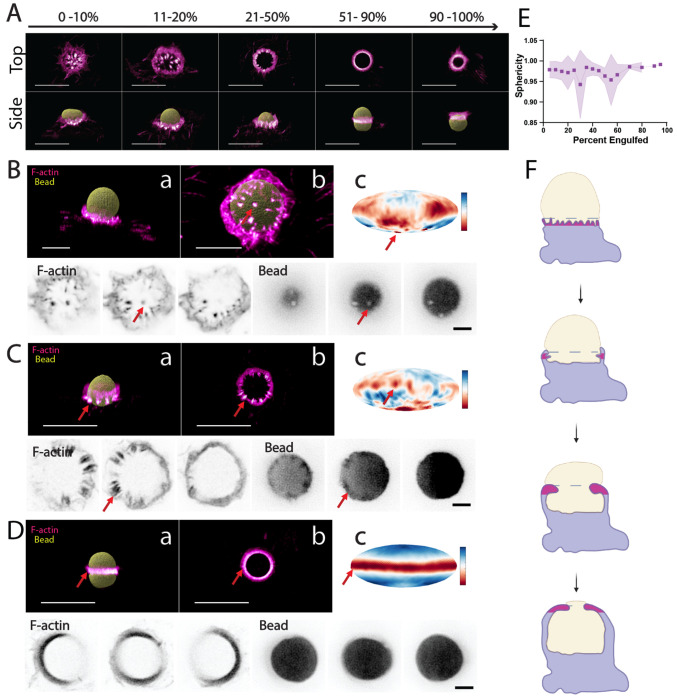
Fixed-cell imaging reveals three distinct types of podosome-like structures during WT phagocytosis. **(A)** Representative phagocytic cups at various stages of engulfment. Top-down (top) and side-view (bottom) images of fixed RAW 264.7 WT macrophages stained with phalloidin (magenta heatmap) show the characteristic organization of F-actin around IgG-coated DAAMPs (yellow) from early attachment (left) to late-stage enclosure (right). Scale bars: 10 μm. (B–D) Montages illustrating basal podosomes (B), actin teeth (C), and the phagocytic ring (D). For each structure: side-view (a) and either bottom-up (for B) or top-down (for C-D) views (b) show F-actin (magenta heatmap) relative to the bead (yellow). Mollweide projections (c; blue-red colormap) depict bead surface proximity to the centroid, with red indicating regions closest to the centroid and blue farthest from the centroid. Bottom panels show representative z-slices from the F-actin (bottom left) and bead (bottom right) channels, with arrows marking the highlighted structures. Scale bars: for B, 5 μm (a,b) and 2 μm (c); for C-D, 10 μm (a,b) and 2 μm (c). **(E)** Bead sphericity changes across phagocytic progression. Sphericity measurements, binned in 5% increments of phagocytic progression (engulfment), show characteristic dips at ~25–30% and ~55–60%, corresponding to bead deformation associated with the actin teeth and the phagocytic ring, respectively (n = 136 beads). **(F)** Schematic of podosome-like actin structures. Diagram highlights the spatial organization of basal podosomes, actin teeth, and the phagocytic ring (magenta) relative to the macrophage (lavender) and bead (beige) throughout phagocytic progression.

To further expand our observations, we used confocal imaging of fixed cells to collect a more extensive dataset of actin organization and target deformations at different stages of cup closure (Fig.4). Using confocal imaging of WT cells fixed during engulfment of DAAMPs, we examined the appearance and frequency of individual podosome-like structures and podosome arrays (ring-like rosettes of podosomes), labeled with phalloidin, at different stages of phagocytosis (Fig.4A). In phagocytic cups corresponding to the stages from the initial bead contact to approximately 20% of bead engulfment (as determined using the secondary antibody staining of the exposed bead surface), we observed distinct basal podosomes that coincided with the sites of bead surface indentation (Fig.4B). At approximately 20-30% cup progression, we observed the presence of podosome-like structures located slightly behind the leading edge of the cup, consistent with the phagocytic actin “teeth” described previously^9^ (Fig.4C). These structures also coincided with inward bead deformations. Finally, we frequently observed phagocytic actin rings/rosettes in many cells that had engulfed 50% or more of the bead (Fig.4D). While the phagocytic rings seemed to be most often present at these stages, there were rare instances where we found rings at earlier stages, with one example found at 12% engulfment. Invariably, the presence of the phagocytic ring correlated with a striking bead deformation. Bead sphericity measurements showed a decrease, indicating a period of heavy deformation, at stages correlating with the appearance of actin teeth (20-30%) and the phagocytic ring (~50%) (Fig.4E).

Together, our observations from the LLSM and confocal imaging of WT RAW264.7 macrophages support a model for phagocytic cup progression where basal podosomes establish initial target contacts, phagocytic actin teeth maintain attachment as the cup advances, and the phagocytic ring/rosette emerges around mid-engulfment (~50%), persisting through closure (Fig.4F).

### Absence of Myo1e/f causes premature formation of the phagocytic actin ring and stalled cup closure

**Figure 5.**
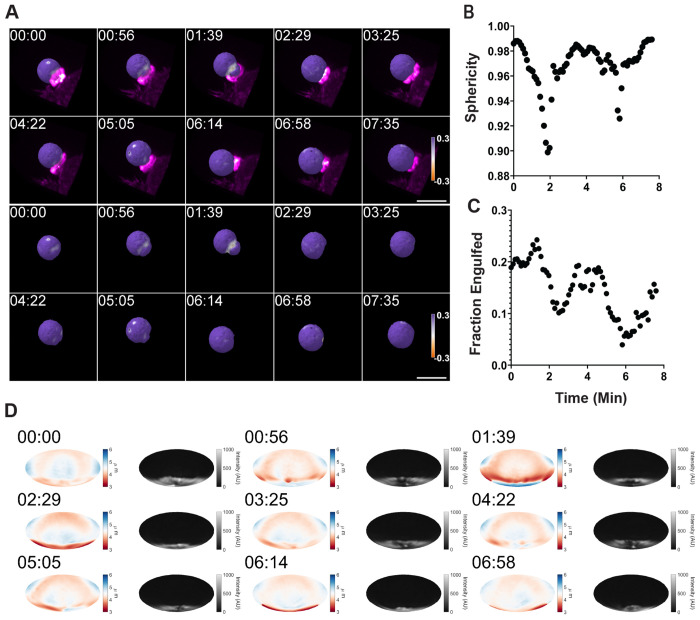
Loss of Myo1e/f leads to premature contraction and stalling of the phagocytic cup. **(A)** Time series of a Myo1e/f dKO macrophage expressing mEmerald–Lifeact (F-actin; magenta intensity map) unsuccessfully attempting to engulf an IgG-coated DAAMP. The DAAMP surface is rendered with a purple-to-orange diverging colormap encoding vertex-based angular curvature derived from the triangulated surface. Lower panels show the bead alone to highlight surface deformation. In this image sequence macrophage attempts several times to engulf the DAAMP, while indenting the particle. Scale bar: 10 μm. **(B)** A graph of sphericity of the bead over time for the event shown in panel A highlights repeated constriction of the cup followed by relaxation. **(C)** Fractional bead engulfment over time for the same event. A drop in bead sphericity precedes the reduction in engulfment fraction, indicating premature contraction and subsequent cup stalling or cup retraction. **(D)** Mollweide projections showing bead surface distance to centroid ranging between 3 μm and 6 μm and corresponding F-actin distribution (right) at each timepoint (excluding the final frame).

After characterizing the typical progression of phagocytosis and F-actin organization in WT macrophages, we next examined Myo1e/f dKO cells to identify alterations that might underlie their reduced phagocytic efficiency. Under identical LLSM conditions, dKO macrophages exhibited a distinctive behavior not observed in WT cells: they frequently formed the phagocytic ring prematurely at an early stage of cup closure, after which the cup receded, and the cell reattempted engulfment (Fig. 5A–D, Fig.S3, Supp. Videos 2,4). In some cases, this sequence occurred multiple times before a bead was successfully internalized. As a result, the complete formation of a phagosome tended to take considerably longer than observed in WT cells.

**Figure 6.**
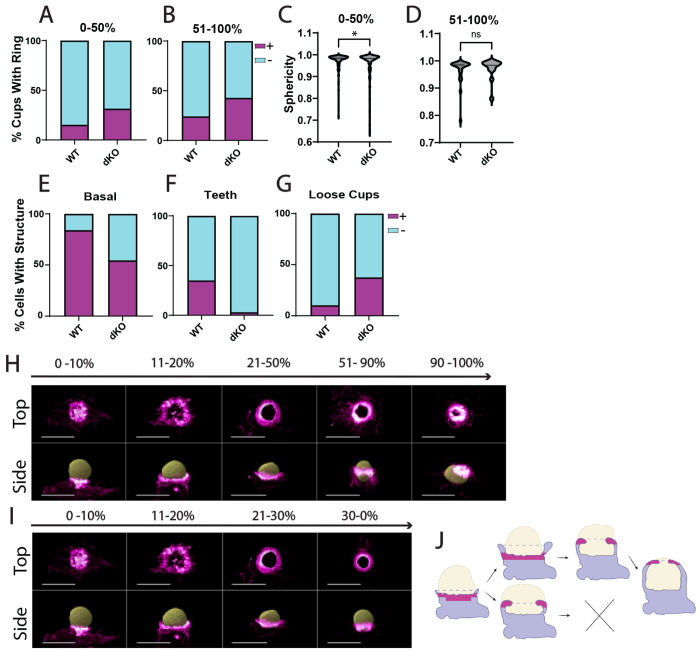
Myo1e/f dKO disrupts the spatiotemporal regulation of F-actin structures during phagocytosis, contributing to delayed or failed particle uptake. (A–B) Frequency of phagocytic ring formation before (A) and after (B) 50% cup closure. Parts-of-a-whole bar graphs show the percentage of fixed RAW 264.7 WT and Myo1e/f dKO macrophages with (magenta) or without (cyan) a phagocytic ring. In dKO cells, the ring appears earlier and more frequently. n = 130 cells per genotype. (C–D) Bead sphericity in fixed cells before (C) and after (D) 50% cup closure. Violin plots reveal a significant decrease in bead sphericity, indicating pronounced deformation, in dKO cups prior to reaching 50% progression. (C) n = 136; (D) n = 29. * p < 0.05. (E–G) Frequency of basal podosomes, actin teeth, and loose cups. Parts-of-a-whole bar graphs show the presence of basal podosomes in cups with ≤ 30% progression (E), and the presence of actin teeth (F) or loose cups (G) across all stages. (E) n = 44; (F–G) n = 160. (H–I) Representative fixed phagocytic cups illustrating successful and failed outcomes in dKO cells. Top-down and side-view images of phalloidin-labeled F-actin (magenta heatmap) and Alexa-647–cadaverine–labeled DAAMPs (yellow) show: (H) Successful fate: phagocytic ring formation in late stages, followed by continued progression. (I) Failed fate: phagocytic ring formation in early stages, associated with subsequent stalling. Scale bars: 10 μm. (J) Schematic of phagocytic fates in Myo1e/f dKO cells. Diagram illustrates podosome structures (magenta) relative to the macrophage (lavender) and DAAMP (beige).

Analysis of fixed dKO macrophages supported these live-cell observations. Among 130 cups examined per cell line, the phagocytic ring appeared more frequently at early stages of phagocytosis in dKO cells (Fig. 6A–B). The phagocytic ring was present in 32% of dKO cups prior to 50% engulfment compared to only 15% in WT cups. Quantitative analysis of bead sphericity across cup stages showed a pronounced reduction in sphericity in dKO cells prior to 50% closure (Fig. 6C–D). This decrease in sphericity likely reflects premature formation of the phagocytic ring and the associated dramatic bead deformation.

Fixed-cell analysis also revealed several additional deviations from the WT phenotype. During early phagocytosis, dKO cells formed distinct basal podosomes at a lower frequency than WT cells (Fig. 6E): 84.1% of WT cups at or below 30% progression displayed distinct basal podosomes compared to 54.5% of dKO cups. Interestingly, some deformation was still detectable at the bead base even in the absence of clear podosome-like structures. “Actin teeth” (individual podosome-like structures located along the cup rim), were rarely present in dKO cups (Fig. 6F), appearing in only 1.9% of dKO cups at any stage compared to 31.6% of WT cups. Consistent with the reduced number of phagocytic podosomes, a greater proportion of dKO cells displayed a loose association with their targets, defined by the observable separation between the actin lamellipodia at the rim of the phagocytic cup and the bead surface (Fig. 6G, Fig.S5). Although this feature was occasionally observed in WT cells, it was much more frequent in dKO cells (38% of dKO cups at any stage compared to 10% of WT cups).

Integrating findings from both LLSM and fixed-cell imaging, we propose a model of phagocytosis in dKO macrophages that involves two distinct fates (Fig. 6H–J). In one outcome, although the cup maintains a loose attachment to the bead, the phagocytic ring forms at an appropriate stage, and the cup progresses to full closure (Fig. 6H). In the alternate fate, the phagocytic ring forms prematurely, and cup progression is stalled (Fig. 6I).

### Myo1e/f localize to the phagocytic ring and podosomes at the base of the phagocytic cup

We next examined the localization of Myo1e and Myo1f within the phagocytic cup to gain insight into how their loss may lead to altered actin organization. Based on the previous reports of Myo1e association with adhesion and actin-rich structures, we hypothesized that both Myo1e and Myo1f would concentrate at the tips of podosomes at the base of the cup and along the leading edge of the cup when the phagocytic ring is present. To test this, we transfected WT and dKO macrophages with mEmerald-tagged Myo1e or Myo1f and performed phagocytosis assays, fixing cells 10 minutes after bead addition and staining F-actin with phalloidin.

**Figure 7.**
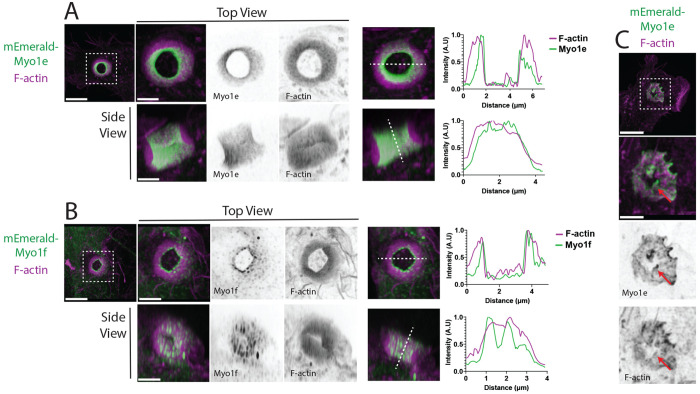
Myo1e and Myo1f localize to key podosome-like F-actin structures within the phagocytic cup. **(A–B)** Myo1e and Myo1f enrichment in the phagocytic ring/rosette. RAW264.7 WT cells expressing mEmerald-Myo1e (A) or mEmerald-Myo1f **(B)** (green) show enrichment along the inner surface of the phagocytic cup. F-actin was labeled with phalloidin (magenta). Shown are 3D projections with the bead excluded for clarity, displayed as topdown views (top) and side views (bottom). Line profiles corresponding to the dotted line are shown at right. Scale bars: main panels, 5 μm; zooms, 2 μm. **(C)** Myo1e localization to basal podosomes in early-stage cups. mEmerald-Myo1e (green) is enriched at the tips of F-actin puncta (magenta) within basal podosomes. Arrow indicates a basal podosome. Scale bars: main panel, 5 μm; zoom, 2 μm.

Confocal imaging revealed that Myo1e and Myo1f exhibited overlapping localization patterns. Both proteins were distributed throughout the inner surface of the phagocytic ring at the membrane–bead interface (Figs. 7A–B) and enriched at the tips of podosomes at the base of the phagocytic cup (Fig. 7C, Fig.S4A). We confirmed previous reports that Myo1e/f localize to the tips of phagocytic actin teeth^9^ (Fig.S4B-C). This localization pattern was consistent in both WT cells and in dKO cells reexpressing either construct, indicating that Myo1e and Myo1f redundantly associate with key actin structures that govern cup progression.

### NM2A localization becomes more diffuse in the absence of Myo1e/f

Given prior evidence suggesting functional interplay between class I myosins and non-muscle myosin IIA (NM2A) during phagocytosis^18^, we next examined how loss of Myo1e/f affects NM2A organization within the phagocytic cup. To visualize NM2 localization, WT and dKO macrophages were transfected with a GFP-tagged myosin regulatory light chain (RLC), which binds NM2 heavy chains, and performed the same phagocytosis assay described above. Cells were fixed 10 minutes after bead addition and stained for F-actin with phalloidin.

**Figure 8.**
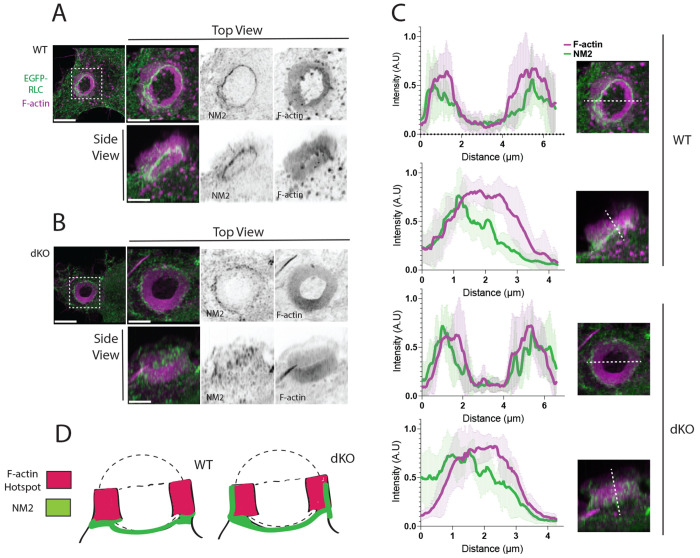
NM2 localization is altered in Myo1e/f dKO phagocytic rings. **(A–B)** NM2 localization relative to F-actin in the phagocytic actin ring in RAW264.7 WT and dKO macrophages. In RAW 264.7 WT macrophages expressing EGFP-Regulatory Light Chain (EGFP-RLC; green), non-muscle myosin II (NM2) forms a concentrated band positioned at the outer edge and base of the phagocytic actin ring (A). F-actin is labeled with phalloidin (magenta). In dKO cells (B), NM2 remains enriched at the outer ring but displays a more diffuse distribution along the vertical axis, rather than the sharply defined band observed in WT cells. Scale bars, 5 μm, zoom scale bars, 2 μm. **(C)** Line profiles of NM2 and F-actin distribution in the phagocytic actin ring. Top-down and side-view intensity profiles were collected from 6 phagocytic rings per cell line and plotted as normalized fluorescence intensity (y-axis) versus position (x-axis, μm), with error bars representing standard deviation from 6 measurements. In top-down views of both WT and dKO rings, NM2 (green) localizes to the outer edges of F-actin ring (magenta). In sideview profiles, WT NM2 signal peaks at the base of the ring and rapidly decreases towards the top. For dKO rings, NM2 localization more closely resembles that of F-actin, with a broad peak of fluorescence intensity. Shaded green or magenta areas indicate the standard deviation for NM2 and F-actin, respectively. **(D)** Schematic of NM2 localization in phagocytic actin rings in WT or dKO macrophages. The phagocytic ring is represented in magenta and NM2 is represented in green, illustrating the spatial restriction of NM2 to the outer- basal band in WT cells and the broader, more diffuse distribution in dKO cells.

In WT macrophages, NM2 localized as a dense band positioned just behind the leading edge of the F-actin–rich cup during most stages of phagocytosis, shifting only after target enclosure (Fig. 8A,C; Fig.S4D-E). NM2 was also enriched at the outer face of the cup, where it did not contact the bead. In contrast, in Myo1e/f- deficient cells, NM2 distribution was markedly altered: the protein appeared diffusely distributed throughout the F-actin–rich region of the cup along the Z-axis rather than forming a discrete basal band (Fig. 8B–C). Despite this difference in vertical organization, the lateral (X-axis) localization along the outer cup surface remained similar to that of WT cells.

These findings indicate that Myo1e/f are required to maintain the spatial distribution of NM2 within the phagocytic cup, suggesting that Myo1e/f help coordinate the balance between contractile and protrusive actin networks necessary for efficient cup progression.

Together, these results reveal that Myo1e and Myo1f play a central role in organizing the cytoskeletal architecture of the phagocytic cup. By localizing to podosomes, actin teeth, and the phagocytic ring, Myo1e/f appear to stabilize the spatial organization of F-actin and constrain NM2 distribution during target uptake. In their absence, loss of podosome integrity, premature formation of the phagocytic ring, and dispersion of NM2 coincide with inefficient or stalled cup progression. These findings suggest that Myo1e/f function as key mechanical and organizational linkers that coordinate protrusive and contractile forces required for successful phagocytosis.

### Loss of Myo1e/f leads to an increase in trogocytosis in RAW 264.7 cells

**Figure 9.**
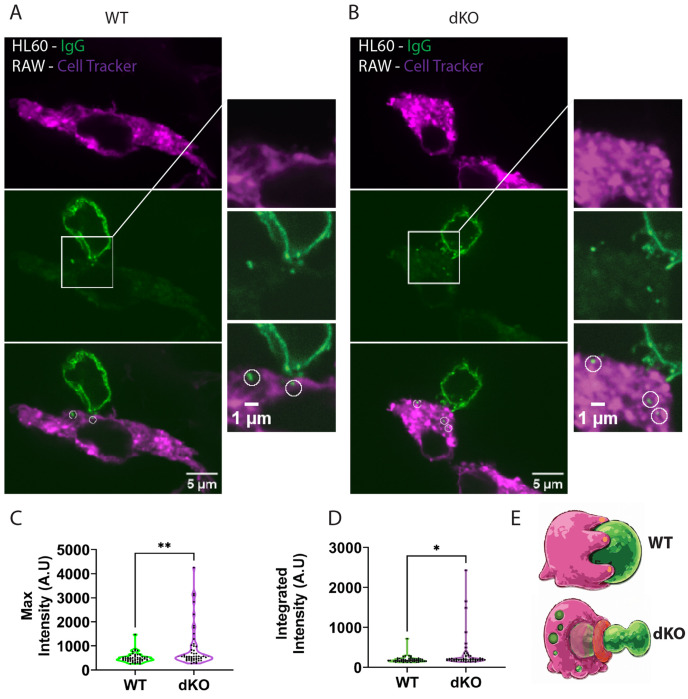
Loss of Myo1e/f increases trogocytosis during antibody-mediated phagocytosis of HL-60 cells by RAW 264.7 macrophages. **(A)** Representative single confocal section from a 3D image stack of a CellTracker-labeled WT RAW 264.7 macrophage (magenta) in contact with an Alexa Fluor 488 anti-biotin-labeled HL-60 cell (green). Boxed region highlights internalized Alexa Fluor 488-positive target fragments within the macrophage cytoplasm. **(B)** Representative single confocal section from a 3D image stack of a CellTracker-labeled Myo1e/f dKO RAW 264.7 macrophage (magenta) in contact with an Alexa Fluor 488 anti-biotin-labeled HL-60 cell (green). Boxed region highlights multiple internalized target-derived fluorescent fragments within the macrophage. **(C)** Quantification of the average maximum fluorescence intensity of 1 μm circular ROIs drawn around internalized target fragments within RAW macrophages. Myo1e/f dKO cells exhibited significantly increased fragment intensity compared with WT cells. Statistical significance was determined by unpaired t-test; P < 0.05. **(D)** Quantification of the average integrated fluorescence intensity of 1 μm circular ROIs drawn around internalized target fragments within RAW macrophages. Myo1e/f dKO cells exhibited significantly increased total fragment fluorescence compared with WT cells. Statistical significance was determined by unpaired t-test; P < 0.005. **(E)** Model illustrating how increased premature cup constriction during early stages of engulfment in Myo1e/f- deficient macrophages may promote pinching and internalization of small target fragments, thereby enhancing trogocytosis (initial drawing created with Google Gemini).

Because loss of Myo1e/f delays phagocytic cup closure and promotes premature cup constriction during early engulfment, we hypothesized that these altered cup dynamics may increase pinching off of small portions of target cells, a process known as trogocytosis^22^. We have previously observed repeated attempts of phagocytes to constrict and ingest small pieces of deformable targets during livecell imaging, however, due to the self-healing nature of DAAMPs, these attempts were not successful. To directly test the trogocytic activity of RAW macrophages, we used an assay in which fluorescent antibody-coated HL-60 cells were presented to Cell Tracker-labeled macrophages (Fig.9). Both WT and dKO RAW cells formed phagocytic cups and attempted to engulf opsonized HL-60 cells. Cells were fixed 30 min after target addition, revealing small AlexaFluor-488-positive HL-60 fragments within macrophages (Fig.9A,B). To quantify trogocytosis, we measured AlexaFluor-488 fluorescence intensity in intracellular HL-60 fragments localized within CellTracker-labeled macrophages, as described in Materials and Methods. Both the maximum fluorescence intensity and total integrated intensity of ingested fragments were higher in dKO macrophages than in WT cells, indicating increased trogocytic activity (Fig.9C,D). These findings suggest Myo1e/f normally suppress excessive cup constriction and stabilize the phagocytic synapse, thereby promoting complete engulfment over partial target removal (Fig.9E).

## Discussion

In this study, we demonstrate that Myo1e and Myo1f are critical regulators of FcR-mediated phagocytosis in RAW 264.7 macrophages. Myo1e/f-deficient macrophages exhibit markedly reduced bead uptake, a phenotype that can be partially rescued by the reintroduction of either myosin, confirming a direct requirement for these proteins in efficient phagocytosis. Using lattice light sheet and confocal microscopy, we identified distinct F-actin structures that appear sequentially during cup progression, including basal podosomes, “actin teeth” (individual podosome-like structures located along the rim of the cup), and a phagocytic ring (a rosette-like array of podosomes). Loss of Myo1e/f disrupts this orderly progression, leading to premature formation of the phagocytic rosette, reduced podosome and actin-tooth formation, and frequent stalling or reversal of cup closure. Together, these results establish a dual role for Myo1e/f in coordinating podosome organization and regulating the balance between protrusive and contractile actin networks required for successful target engulfment.

Our findings indicate that Myo1e/f contribute to phagocytosis through two complementary mechanisms. First, they regulate the spatiotemporal organization of podosome-like structures within the phagocytic cup, promoting efficient target adhesion and the coordinated formation of protrusive structures such as actin teeth. Second, they help balance the generation of protrusive and contractile forces within the cup, ensuring smooth progression and closure. In the absence of Myo1e/f, this balance shifts from the formation and maintenance of individual podosome-like structures to premature assembly of podosome rosettes/rings, resulting in stalled or aborted phagocytic events, likely due to excessive contractile forces exerted prior to 50% engulfment. Together, these observations suggest that Myo1e/f act as key organizers of cytoskeletal transitions during target engulfment.

Our results extend our previous observations that identified Myo1e/f-positive actin teeth and myosin-II–dependent constriction as key force-generating mechanisms during phagocytosis and implicated Myo1e/f in phagocytic cup closure^8,18,23^. Using microparticle traction force microscopy and WT RAW cells we have previously demonstrated that actin teeth, enriched in Myo1e/f, exert normal/protrusive forces on the target surface, and that target deformation by protrusive forces is reduced following Arp2/3 inhibition^9^. Primary mouse BMDMs also exhibit protrusive forces leading to phagocytic target deformation, however, the baseline cup architecture differs between macrophage systems: in WT RAW cells, F-actin is concentrated predominantly at the cup rim in the form of discrete teeth^8^, whereas in WT BMDMs it is distributed more broadly throughout the cup^18^.

Importantly, earlier work^10^ showed that during frustrated (planar) phagocytosis the region of closest apposition to the IgG-coated surface coincides with a ring of podosome-like structures rather than the leading edge of the cell and proposed that these actin-rich structures help maintain close membrane–target contact. Consistent with and extending this idea, Myo1e/f-deficient RAW cells examined in the current study formed markedly fewer individual teeth (and more pronounced podosome rosettes), displayed looser attachment to their targets, yet retained the ability to deform beads through constriction of the phagocytic rim. Together, these findings suggest that the principal role of actin teeth may be less in bulk target constriction than previously emphasized, and more in maintaining close membrane–target apposition and stabilizing productive protrusive adhesions during cup extension. Loss of Myo1e/f may impair organization of these structures, biasing the cup toward merging of individual podosome-like structures into rosettes and premature constriction.

This interpretation is supported by recent work from Sopelniak et al^24^, which showed that disrupting podosome assembly via inhibition of Arp2/3 or Src reduced phagocyte-target attachment and impaired overall uptake efficiency and phagosome maturation during uptake of yeast *Candida auris*. Together, these studies highlight a conserved role for podosome-like protrusions in maintaining mechanical coupling between the macrophage and its target. Our data further identify Myo1e/f as key regulators of this coupling, coordinating podosome formation and timing to sustain adhesion and promote efficient cup progression.

Once actin-rich phagocytic rings were formed, we observed that Myo1e/f localized throughout the inner surface of the phagocytic ring, suggesting that this structure represents a podosome-derived superstructure similar to the podosome rosettes found on ventral cell surfaces during cell migration, matrix degradation, and frustrated phagocytosis^25–29^. Because Myo1e/f are well-established markers of podosomes, their enrichment supports the idea that the ring is composed of interconnected podosome-like units adapted for the three-dimensional geometry of phagocytic cups. Unlike the ventral podosome rosettes examined by Ouderkirk and Krendel^28^, in which Myo1e/f localize to the outer edge of the adhesion, in phagocytic podosome rings these proteins occupy the inner, bead-facing surface, an inversion consistent with the reversed membrane curvature during target engulfment. While in ventral rosettes these myosins face the cell-matrix interface, in the phagocytic cup they are oriented toward the target-facing invagination, effectively ‘pinning’ the membrane to the bead from the inside out.

Our findings also suggest that phagocytic ring formation and contractile constriction are not, by themselves, sufficient to maintain continuous close membrane–target apposition. Although the phagocytic ring can generate substantial circumferential forces and deform the target, such forces may operate at the mesoscale, effectively tightening the purse-string around the target, but without the local anchoring provided by actin teeth. Without the local anchors, the membrane may fail to follow the precise topography of the target, particularly if increased cortical tension during constriction tends to pull the membrane taut. Thus, actin teeth/podosome-like structures may provide a distinct adhesive-protrusive function, acting as discrete sites that maintain intimate membrane–target contact, sustain receptor engagement, and stabilize productive cup advancement. This interpretation is consistent with prior observations in frustrated phagocytosis. Myo1e/f loss may uncouple ring constriction from local adhesive apposition, allowing the cup to retain rim-dominant contractile deformation while losing the continuous target contact normally supported by actin teeth.

In WT macrophages, NM2 formed a condensed band just below the phagocytic ring, consistent with the presence of linear actin filaments interconnecting the podosome units, as described in frustrated phagocytosis models^10^. The colocalization of Myo1e/f and NM2 within this region indicates that the phagocytic ring can integrate both protrusive and contractile functions. Such dual-force generation may explain why phagocytosis can proceed, albeit less efficiently, when either Myo1e/f or NM2 activity is impaired, as each system may partially compensate for the other to drive target constriction and closure.

The diffuse redistribution of NM2 observed in Myo1e/f-deficient macrophages provides a potential explanation for the stalled cup progression captured in our LLSM imaging. Van Zon et al^30^ proposed a mathematical model in which phagocytic cup extension halts when the maximum force generated by the actin network fails to exceed the force required to advance the cup around the widest point of the target. Our findings are consistent with such a scenario, although our interpretation differs. We propose that, in WT cells, contractile structures assemble after approximately 50% engulfment, where they cooperate with protrusive actin teeth to promote coordinated cup closure. In contrast, in Myo1e/f-deficient cells, contractility appears prematurely and becomes spatially disorganized. Simultaneously, loss of Myo1e/f impairs actin teeth formation or maintenance and reduces protrusive stabilization of the advancing cup. Thus, contractile forces may be sufficient to deform or locally pinch the target membrane yet insufficiently coordinated to drive continued cup advancement around the particle. The combined loss of protrusive support and organized constriction could therefore produce the repeated initiation and retraction cycles characteristic of Myo1e/f-deficient cells while favoring partial target scission events.

Consistent with this model, Myo1e/f-deficient macrophages exhibited increased trogocytosis of opsonized HL-60 targets. Our observations show that both WT and dKO macrophages were actively engaged in trogocytosis, similarly to the observation of Rollins et al. using BMDMs engineered to phagocytose cancer cells^31^. dKO cells accumulated greater amounts of internalized target fragments than WT macrophages, indicating a shift toward partial target acquisition. We propose that premature, poorly coordinated rim constriction combined with reduced protrusive stabilization favors pinching and scission of small membrane fragments instead of progressive cup extension around the entire target. In this framework, Myo1e/f normally promote productive phagocytosis not only by supporting cup closure, but also by suppressing unproductive fragmentary ingestion events. Given the prevalence of trogocytosis in 3D environments^31^ and its role in affecting the efficacy of CAR-T cancer therapies^32^, the role of Myo1e/f in trogocytosis regulation in complex tissue environments will need to be further investigated in future studies.

Premature or poorly coordinated formation of contractile structures in Myo1e/f-deficient macrophages may arise from dysregulated NM2A localization or activation caused by loss of Myo1e-dependent signaling and/or altered adhesion architecture. Disruption of small podosome-like adhesion structures could remove local mechanical or biochemical cues that normally constrain NM2A activity to the appropriate stage and region of the cup. While the specific pathways linking Myo1e and NM2A have not been fully elucidated, Myo1e has previously been shown to interact with LSP1 at sites of phagocytosis, forming an LSP1–Myo1e complex that promotes cup progression^33^. LSP1 also regulates the distribution and activity of NM2A at podosomes^34^. Thus, Myo1e–LSP1 interaction provides one possible pathway, while loss of podosome-like adhesion itself provides an additional non-mutually exclusive mechanism for NM2A misregulation in the absence of Myo1e/f.

Together, our findings establish Myo1e and Myo1f as central coordinators of cytoskeletal remodeling during FcR-mediated phagocytosis. By regulating the formation and timing of podosome-derived actin structures, these myosins maintain the balance between protrusive and contractile forces required for efficient target engulfment while limiting diversion into partial ingestion pathways such as trogocytosis. A deeper understanding of how unconventional myosins integrate with signaling and adhesion networks will be essential for elucidating the molecular logic of phagocytosis. Future work should focus on identifying binding partners of Myo1e/f and defining how these interactions influence actin dynamics, adhesion turnover, and mechanosensation within the cup. Because phagocytosis is inherently mechanosensitive and Myo1e localization has been shown to respond to membrane tension changes^35^, exploring how substrate stiffness or target rigidity modulates Myo1e/f function may reveal broader principles governing myosindependent force coordination in immune cells. Ultimately, these insights will advance our understanding of how the actin–myosin machinery supports immune surveillance and how its dysregulation contributes to pathologies involving defective phagocytosis.

## Materials and Methods

### Cell Culture:

Cells (RAW 264.7 and BMDMs) were cultured in high glucose (4.5g/L) Dulbecco’s media (DMEM) (Fisher, MT10013CV) with 10% non-heat inactivated fetal bovine serum (Fisher, FB12999102) and 1% penicillin/streptomycin (Gibco, 152240062) and incubated at 37°C with 5% CO2. Cultures were maintained in tissue culture-treated 100mm dishes. Besides instances where the cells were to be used for transfection, cells were detached from the plates with Trypsin-EDTA 0.25% (Gibco, 25200056).

HL-60 cells were cultured in glutamine-supplemented RPMI-1640 (Cytiva, SH30027.01) with 20% non-heat-inactivated fetal bovine serum (Fisher, FB12999102) and 1% antibiotic-antimycotic (HyClone, SV30079.01) and incubated at 37°C with 5% CO2. Cultures were maintained in tissue culture-treated 100mm dishes below 1x10^6^ cells per mL.

### Western blotting:

Cell lysates were generated using cold immunoprecipitation (IP) lysis buffer (50 mM Tris/HCl, pH 7.5; 150 mM NaCl; 1 mM EDTA; 1% Triton X-100 (w/v)) with added protease inhibitor (Thermo Fisher, PI88266) and phosphatase inhibitor (Roche, 04906837001). Cells were scraped into the lysis buffer, mixed with a 5X SDS-PAGE sample buffer (50% glycerol, 25% β-mercaptoethanol, 15% SDS, 0.025% bromophenol blue 250 mM Tris–HCl, pH 6.8), and boiled for 5 min. Samples were separated on an SDS-PAGE gradient gel (7.5%–15% polyacrylamide) and transferred to PVDF membrane (EMD Millipore, IPVH00010). Total protein staining was performed using SYPRO Ruby blot stain (ThermoFisher) according to manufacturer’s instructions. Membrane was blocked with 5% BSA in TBST (20 mM Tris–HCl, pH 7.5, 150 mM NaCl, 0.1% Tween 20) and the membrane was incubated with primary antibodies. After incubating the membrane with HRP-tagged secondary antibodies, blot was developed using ECL reagent (BioRad, 1705060). The ECL signal was visualized using a Chemidoc MP imaging system (Bio-Rad) and Image Lab analysis software (Bio-Rad). The following primary antibodies were used: rabbit anti-myo1e (Sigma-Aldrich, HPA023886, Fig. S1A), homemade rabbit anti-myo1e (RRID AB_2909514, Fig. S1B), mouse anti-myo1f (Santa-Cruz, sc-376534).

### Macrophage Preparation:

All experiments involving mouse bone marrow isolation were performed according to the animal protocol approved by the Upstate Medical University IACUC. Myo1e and Myo1f KO mice were maintained on the C57Bl/6 background and all experiments used age- and sex-matched bone marrow preparations. BMDM isolation and differentiation has been previously described^18^. Briefly, bone marrow cells were thawed from stocks stored in liquid nitrogen that were frozen after the initial extraction from mouse femurs. They were then maintained in DMEM with 10% FBS and 20 ng/mL M-CSF at 37°C. The media was refreshed with fresh M-CSF-containing media at 3 days post thaw and media lacking M-CSF at 7 days post thaw.

RAW264.7 macrophages (ATCC TIB-71) were thawed from liquid nitrogen stock. They were re-passaged 3 days post thaw before use in any assays. WT cells used in this manuscript range from passage 7 to passage 10, while myo1e/f dKO cells range from passage 18 to passage 23. Myo1e/f dKO cells were generated by Crispr-mediated gene editing (Fig.1) by Synthego and subjected to limited-dilution cloning.

### Transfection:

Plasmid DNA was transfected into RAW264.7 cells using the Neon electroporation system (ThermoFisher, MPK10096). Prior to transfection, cells were detached using TrypLE Express (ThermoFisher, 12604021) and brought to a concentration of 4 x 10^5^ cells per experimental well. Cells were then centrifuged (300 x g, 5 min) and resuspended in 100 μL of cold R-buffer (part of MPK10096 kit) per experimental well. Plasmid DNA was then added at a concentration of 15 ug per experimental well. Cell/DNA solutions were then electroporated with the following settings: one 1720V pulse for 20 ms. They were then plated onto an acid washed, 18 mm, glass coverslip in the appropriate well of a 12 well dish with 1 mL of DMEM with 10% heat inactivated FBS (R&D Systems, S11150) without antibiotic/antimycotic. Plates were incubated at 37°C for 24 h prior to use.

### Bead Functionalization:

9 μm carboxylate-modified polystyrene beads (Bangs lab, PC07001) and deformable acrylamide-co-acrylic acid microparticles (DAAMPs, 8.8 μm in diameter and ~1 kPa in stiffness)^19^ were coated with cadaverine-conjugated TetramethylRhodamine (TRITC) (Life technologies, A1318) or AlexaFluor-647 (Thermo Fisher, A30676) and Bovine Serum Albumin (BSA) (Sigma-Aldrich, A3059) for use in phagocytosis assays. Beads were functionalized as in Vorselen et al 2020^19^. Briefly, beads were diluted to 5% (v/v) concentration and washed twice in activation buffer (100mM MES, 200mM NaCl, pH 6.0) using resuspension and centrifugation (11,000 x g, 5min). They were then mixed at room temperature for 15 minutes in activation buffer supplemented with 40mg/mL 1-ethyl-3-(3-dimethylaminopropyl) carbodiimide, 20 mg/mL N-hydroxysuccinimide (NHS), and 0.1% (v/v) Tween 20. Following activation, beads were pelleted and washed three times in PBS pH 8.0 (adjusted with NaOH) with 0.1% Tween 20. Immediately following the final wash, the beads were resuspended in PBS pH 8.0 containing 20 mg/mL BSA and rotated for 1hr at room temperature. Then cadaverine conjugate was added to a final concentration of 0.2 mM and beads were rotated for an additional hour. Unreacted NHS groups were blocked with 100mM TRIS and 100mM ethanolamine (pH9.0) for 30 minutes with mixing. The beads were then washed three times in PBS, pH 7.4 without Tween 20 and resuspended in the same buffer for storage at 4°C. The day before use, beads were washed twice in sterile PBS and opsonized with 3mg/mL rabbit anti-BSA antibody (MP Biomedicals, 651111) while rotating at 4°C overnight. Beads were then washed three times in PBS (with centrifugation at 3000 x g, 5 min) and resuspended in DMEM without serum or antibiotics.

### Phagocytosis Assay:

Cells were plated on 18mm acid-washed glass coverslips in 12-well plates at a density of 2.5 * 10^5^cells/well one day before the experiment. For experiments involving transfection, cells were electroporated at the time of plating using the Neon electroporation system (see Transfection section for details). On the day of the experiment, IgG-coated beads were washed three times with PBS and resuspended in DMEM at a concentration of 2 beads/cell in a final volume of 1 mL per well. The medium in each well was then replaced with the bead suspension. In phagocytic efficiency assays utilizing Halo-tagged constructs, cells were suspended in DMEM with added serum and 0.25 μM JF646 Halo-ligand^36^ for one hour prior to bead suspension. To synchronize phagocytosis across wells, plates were centrifuged at room temperature for 3 minutes at 300 x g to sediment beads onto the cell surface. Plates were then incubated at 37°C for indicated times. Following incubation, cells were fixed by adding 1 mL of 8% paraformaldehyde (PFA) solution into each well (final concentration 4%) for 10 minutes. Wells were then washed three times with PBS for 5 minutes each.

### Phagocytic E6iciency:

Following the procedure outlined in the Phagocytosis Assay section, DyLight-488-conjugated goat-anti-rabbit secondary antibody (Fisher, P35552) (1:1000 dilution in PBS) was added to each well for 1 hour. After the antibody incubation, wells were washed three times in PBS for 5 minutes each. The coverslips were then mounted onto glass microscope slides using ProLong Gold (Thermo-Fisher, P36930) or ProLong Glass (Thermo-Fisher, P36980) anti-fade mounting media.

### Fixed-Cell Phagocytic Cup Staining:

Following the procedure outlined in the Phagocytosis Assay section, Alexa-405-conjugated goat-anti-rabbit secondary antibody (Thermo-Fisher, A31556) (1:750 dilution in PBS) was added to each well for 1 hour to label exposed bead surface. Following antibody incubation, the wells were washed 3x in PBS for 5 minutes each. Then Triton X-100 0.25% (v/v) in PBS was added to each well for 3 minutes for permeabilization. The Triton X-100 solution was removed, and 1mL of Alexa-568 Phalloidin (Invitrogen, A12380) (1:1000 dilution in PBS) was added to each well for 1 hour. This was followed by washing with PBS three times for 5 minutes each. The coverslips were then mounted onto glass microscope slides using ProLong Gold or ProLong Glass anti-fade mounting media.

### Trogocytosis Assay:

Cells were plated on 18 mm glass coverslips in 12-well plates one day prior to the experiment and maintained in complete growth medium. On the day of the assay, RAW 264.7 macrophages were washed three times with PBS and incubated in DMEM containing 24 μM CellTracker CM-DiI for 90 min to label the macrophage cytosol. Cells were then washed three times with PBS, replenished with complete medium, and returned to 37°C with 5% CO_2_ until use.

HL-60 target cells were collected by centrifugation at 200 × g for 3 min, washed in PBS, and resuspended in PBS containing 0.1 M sodium bicarbonate. For biotinylation, 4 × 10^6^ cells were incubated with 6 μL of 292 mM NHS-biotin for 30 min at room temperature. Cells were then washed three times by centrifugation (200 × g, 3 min) and resuspended in PBS. Biotinylated HL-60 cells were labeled by incubation with Alexa Fluor 488 anti-biotin antibody (40 μL of 200 μg/mL stock) for 1 h at 4°C, followed by three additional washes in PBS.

For trogocytosis assays, 1 × 10^6 labeled HL-60 cells were added to each coverslip containing CellTracker-labeled RAW macrophages. Plates were centrifuged at 200 × g for 3 min to synchronize target-cell contact and uptake, then incubated at 37°C with 5% CO_2_ for 30 min. Following incubation, cells were washed three times with PBS and fixed in 4% paraformaldehyde for 15 min at room temperature. Coverslips were then mounted onto glass slides for imaging.

For quantification, 3D image stacks were manually analyzed in ImageJ. Only cell pairs consisting of a single RAW macrophage in contact with a single HL-60 cell, where the macrophage had not fully engulfed any targets, were included. Alexa Fluor 488-positive signal localized within the CellTracker-positive macrophage volume and spatially separable from the main HL-60 cell body was scored as a trogocytic fragment. A 1 μm circular region of interest was drawn around each fragment in corresponding z-planes, and maximum and integrated fluorescence intensity values were recorded for analysis.

### Lattice Light-Sheet Microscopy:

LLSM experiments were performed at the Advanced Imaging Center at the HHMI - Janelia Research Campus. RAW264.7 macrophages were cultured on 5 mm round coverslips (Warner Instruments Cs-5R). Coverslips were then mounted on the LLSM sample holder and submerged in FluoroBrite medium (ThermoFisher, A1896701) supplemented with 10% fetal bovine serum and 1% Antibiotic-Antimyocotic. Imaging medium was maintained at 37°C and 5% CO2 using a custom incubation system (Okolab). The design of the LLSM is as previously described^37^. Imaging was performed using a square lattice pattern (NA max: 0.4; NA min: 0.325), a Special Optics 0.65 NA, 3.75 mm working distance water-dipping excitation objective, and 488 nm and 647 nm excitation laser lines (100 mW base power, 4% - 20% acoustooptic tunable filter transmission). Volumetric images (512 x 1024 pixel field of view (FOV) with 201 z steps) were collected while scanning the sample stage laterally at an angle of 31.8° relative to the optical axis of the detection objective (Nikon CFI Apo LWD 25x– Water dipping, 63x magnification, 1.1 NA, 3 mm working distance) with a step size of 0.4 μm and an exposure time of 10 ms. Stacks were collected every 6.24 seconds until manually stopped for each experiment based on the duration of the particular phagocytic event being observed. Emitted fluorescence was filtered through two notch filters (Semrock NF03-488E-25 and NF03-642E-25) and imaged onto a Hamamatsu ORCA-Flash 4.0 v2 sCMOS detector. After data collection, images were deskewed and deconvolved (10 iterations of Richardson-Lucy deconvolution with an experimentally determined point spread function) using a custom analysis pipeline^38^. The final voxel size after processing was 0.104 × 0.104 × 0.211 μm.

### Confocal Microscopy:

Confocal images were collected using the Nikon Eclipse Ti2e Spinning Disk Confocal Microscope equipped with a CSU-X1 (Yokogawa) with an array of 50-micron pinholes with a spacing of 250 microns. The microscope was equipped with a filter wheel with the following filters: ET455/50M (Chroma), ET525/36M (Chroma), ET605/52M (Chroma), ET705/72M EM (Chroma). Sample excitation was achieved using a LUNF-XL (Nikon) laser combiner and an iChrome MLE-LFA-NI2 (TOPTICA Photonics), 4 laser line source with 405 nm, 488 nm, 561nm, and 640 nm excitations. Fixed cell phagocytic cup images were acquired using the CFI60 PLAN APOCHROMAT LAMBDA D 100X Oil (Nikon) objective lens with type F immersion oil (Nikon, MXA22168). Images were captured using an Orca FusionBT (Hamamatsu) camera with an active chip size of 16.43 x 16.43 microns. Z-stacks were acquired with a NIDAQ Piezo Z device at 0.2μm steps. Z-stacks were collected starting at the ventral-most surface of the cell to the ventral-most surface of the bead. Automatic deconvolution of images based on microscope specific processing was completed immediately after initial acquisition using NIS Elements Advanced Research version 5.42.02.

### DIC and Wide Field (WF) Fluorescence Microscopy:

DIC and WF images for the phagocytic efficiency assays were obtained using a Nikon Eclipse TE2000-E2 Microscope system equipped with a Photometric Prime 95B scientific CMOS camera. Images were acquired using a 20x magnification, Plan Apo, WD 1.0, NA 0.75 objective, using NIS-Elements advanced research software version 4.60.00 64-bit.

### Phagocytic E6iciency:

Phagocytic efficiency data was analyzed using FIJI software (RRID:SCR_002285). Images were channel separated and the corresponding information (cells (C_t_), total beads (B_t_), unengulfed beads (B_u_), phagocytic cells (C_p_), cells expressing plasmid construct (C_e_)) were counted using the FIJI cell counter plug in. DIC images were used to count cells, 561 nm emission images were used to count total beads, 488 nm for unengulfed beads, 640 nm for plasmid construct expression, and 4 channel merged images to count phagocytic cells. Ten 660 x 660 μm fields of view (FOV) were collected and analyzed per experimental well in each experiment. The reported data are based on the sum of counts from all ten FOV. Calculations were made as follows:

Eq.1 %Phagocytosis=(1−(BuBt))×100

Eq.2 Expression=CeCt

Figures were made using GraphPad Prism software (RRID_SCR_002798). Statistical calculations were performed using in-built analysis operations. In both Figure.2C and 2D a two-way ANOVA was used with post-hoc comparison tests (uncorrected Fisher’s LSD for 2C and Dunnett’s multiple comparisons in 2D) with an alpha of 0.05.

### ChimeraX data visualization:

Deconvolved image stacks were visualized in UCSF ChimeraX^39,40^. From the volumetric intensity data, 3D bead surfaces were rendered as either wire-frame meshes or opaque isosurfaces to observe IgG-coated beads undergoing phagocytosis. Surface curvature was quantified using the ChimeraX *Measure Convexity* function, which reports surface curvature derived from the triangulated surface geometry per vertex. Local curvature values (steradians) were averaged across groups of 10 neighboring vertices to reduce noise and used to generate color-mapped isosurfaces as needed. Additionally, the visualization of adjacent cellular components, such as Lifeact-labeled actin filaments, was performed using maximum intensity projections (MIPs) along the viewing axis.

For preparation of representative time-lapse images and 3D renderings, selected regions of interest were processed in UCSF ChimeraX. The volume region and erase functions were used to restrict displayed volumes to regions containing the cells and DAAMPs of interest. The surface dust function was applied to remove small disconnected voxel clusters arising from background noise in isosurface renderings. These processing steps were used for visualization purposes only and were not applied to raw data used for quantitative analysis. Figures subjected to cropping and/or surface dust filtering include Figs. 3, 5, Supplementary Figs. 2 and 4, and associated supplementary videos.

### DAAM Particle Analysis

Deconvolved images from LLSM and confocal microscopy images were analyzed using custom MATLAB functions adapted from Vorselen et al^9^. Briefly, image stacks of antibody-coated beads were reconstructed in 3D using particle edge coordinates determined with sub-pixel accuracy through the Gaussian fitting of radial intensity profiles as previously described^9,19^. Prior to reconstruction, image stacks were smoothed to reduce high-frequency noise. The resulting particle surface was resampled with approximately equidistant spacing between neighboring vertices with a separation distance of 250 nm. From the reconstructed triangulation of the particle, we compute its triangulated surface area, S, and volume, V, for subsequent calculation of particle sphericity, Ψ given by the following equation,

Eq. $$\Psi =\frac{\pi^{1/3}\;*\;{\left(6*V\right)}^{2/3}}{S}$$

Lastly, the fraction of the particle engulfed is determined through mapping the signal of the fluorescently-stained free (non-engulfed) particle surface onto the reconstructed edge coordinates. To do so, maximum-intensity projections of the smoothed free-surface stain channel (smoothing factor = 0.5), were registered to the triangulated particle’s edge coordinates within a tolerance of 1 μm, followed by masking as described by Vorselen et al^19^.

### Fixed Cup Structural Analysis:

Data for Figure 6 A,B,E,F, and G were gathered by manual scoring by an observer blinded to the sample identity using qualitative pre-defined criteria from 3D reconstructed cups visualized in both FIJI and ChimeraX software. Cells were scored positive for phagocytic rings when a circular band of highly enriched F-actin signal was observed in the phagocytic cup. Basal podosomes were defined as high-intensity F-actin puncta at the bottom of the cup that measured from 0.25-1.0 μm in diameter using a line scan. If a cup had at least one punctum that met the above criteria, it was marked as positive for basal podosomes. Phagocytic teeth were defined as distinct, high-intensity F-actin signals within the body of the phagocytic cup which protruded into the bead-containing region. Loose cups were defined as phagocytic cups which had the presence of secondary antibody signal below the leading edge of F-actin at the rim of the cup. Graphs were made using GraphPad Prism software.

## Supplementary Material

Supplement 1

Supplement 2

Supplement 3

Supplement 4

5
