# Supplementary material for: Myo1e/f regulate phagocytic podosomes to promote efficient cup closure in macrophages
Source: bioRxiv. 2026 May 1:2026.04.30.721640. Preprint. [Version 1] doi: 10.64898/2026.04.30.721640 (PMC13142426; doi:10.64898/2026.04.30.721640)
Supplement: 5 [file NIHPP2026.04.30.721640v1-supplement-5.pdf]

bioRxiv preprint doi: https://doi.org/10.64898/2026.04.30.721640; this version posted May 1, 2026. The copyright holder for this preprint (which was not certified by peer review) is the author/funder, who has granted bioRxiv a license to display the preprint in perpetuity. It is made available under aCC-BY 4.0 International license.

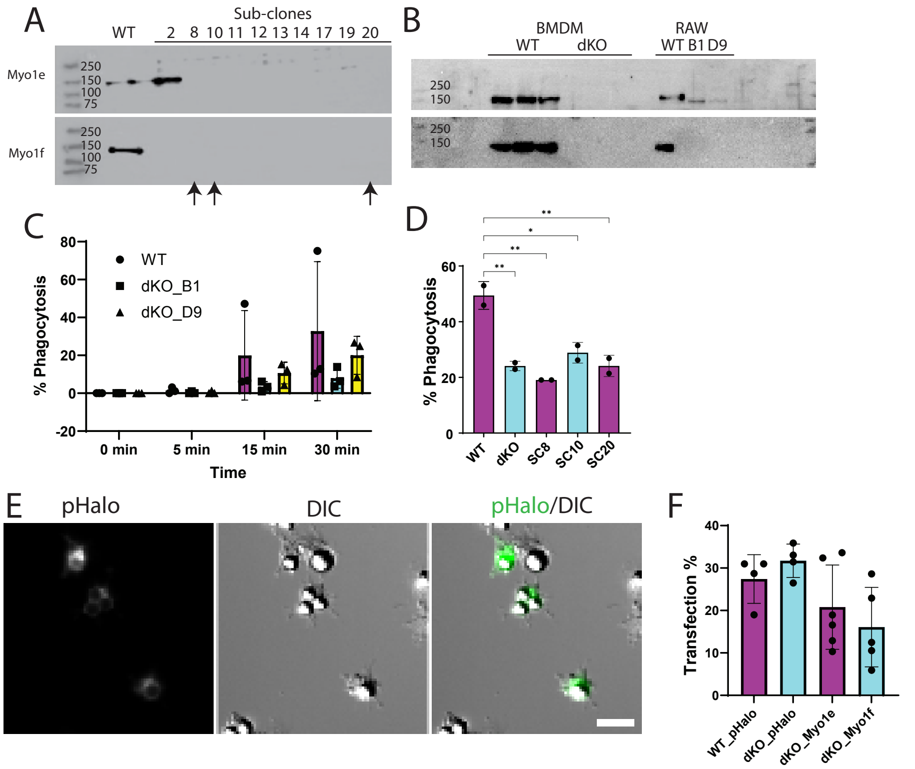

Supplemental Fig. 1. Isolation and testing of additional dKO clones. **(A)** Analysis of additional subclones of dKO RAW264.7 macrophages using anti-Myo1e or anti-Myo1f Western blotting. Several monoclonal cell populations, subcloned by dilution from a CRISPR-edited RAW264.7 pool, were examined for the presence of Myo1e or Myo1f using antibodies HPA023886 for Myo1e and sc-376534 for Myo1f. Arrows indicate three clones that were used in additional phagocytic efficiency experiments in panel D. **(B)** Anti-Myo1e or anti-Myo1f Western blot analysis of the two dKO clones provided by Synthego. The Myo1e/f expression in two RAW264.7 dKO clones produced by Synthego (B1 and D9) was compared to RAW264.7 WT as well as BMDM WT and dKO cells. **(C)** Time-course of phagocytic uptake comparing RAW264.7 WT and the two Synthego-generated dKO clones. Both B1 (cyan bar, square marker) and D9 (yellow bar, triangle marker) display reduced phagocytic uptake of IgG-coated polystyrene beads at 15- and 30-minute time points when compared to WT (magenta bar, circle marker), but D9 shows a less severe reduction. N = 3. **(D)** Phagocytic efficiency of additional sub-cloned Myo1e/f dKO RAW264.7 macrophage populations at 30-minute time point. The three dKO populations from panel A were compared to the WT and dKO (clone B1) macrophages. Each dKO population showed a reduction of phagocytic uptake when compared to the WT. * = $P<0.05$, ** = $P<0.01$. N = 2. **(E)** Montage illustrating transfection of RAW264.7 macrophages with pHalo-tagged constructs for phagocytic efficiency experiments shown in Fig.2D. Scale bar, 25µm. **(F)** Cells expressing pHalo-tagged empty-vector or Myo1e/f constructs were identified by comparing the amount of cells expressing pHalo signal to the total amount of cells.

bioRxiv preprint doi: https://doi.org/10.64898/2026.04.30.721640; this version posted May 1, 2026. The copyright holder for this preprint (which was not certified by peer review) is the author/funder, who has granted bioRxiv a license to display the preprint in perpetuity. It is made available under aCC-BY 4.0 International license.

Percent of cells expressing pHalo signal in each cell line used in Figure.3D.

bioRxiv preprint doi: https://doi.org/10.64898/2026.04.30.721640; this version posted May 1, 2026. The copyright holder for this preprint (which was not certified by peer review) is the author/funder, who has granted bioRxiv a license to display the preprint in perpetuity. It is made available under aCC-BY 4.0 International license.

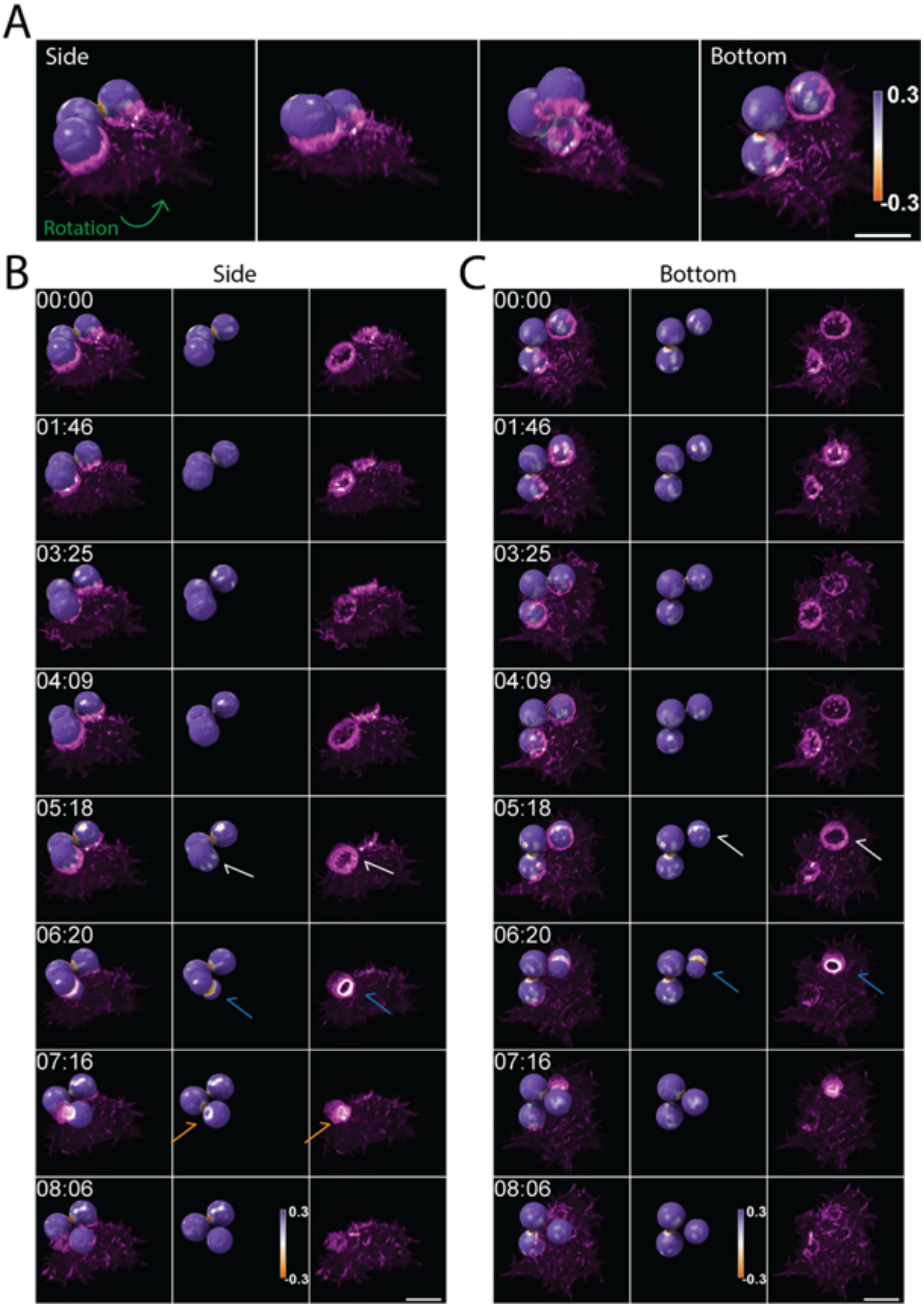

bioRxiv preprint doi: https://doi.org/10.64898/2026.04.30.721640; this version posted May 1, 2026. The copyright holder for this preprint (which was not certified by peer review) is the author/funder, who has granted bioRxiv a license to display the preprint in perpetuity. It is made available under aCC-BY 4.0 International license.

Supplemental Fig. 2 (related to Fig. 3): Additional example of actin-driven particle deformation during FcγR mediated phagocytosis

(A) The initial frame of an LLSM time-lapse movie showing WT RAW 264.7 macrophages expressing mEmerald-Lifeact engaging several antibody-coated DAAMPs. Lifeact signal is displayed as a magenta-to-white MIP, while DAAMP surfaces are rendered as opaque isosurfaces with a diverging color palette encoding local surface curvature (purple to orange, with white corresponding to zero curvature). The montage shows three progressively rotated side views of the same field of view (left to right), followed by a bottom view.

(B-C) Individual frames from the same time series show two different viewing angles of the WT macrophages internalizing antibody-coated DAAMPs. During particle uptake, actin dynamics associated with phagosome formation include the appearance of podosome-like structures that induce local indentations in the DAAMP surface (white arrows). These podosome-like structures subsequently coalesce into a ring-shaped structure resembling a podosome rosette (blue arrows), which induces deep indentations in the DAAMP. During later stages of internalization, actin pools into a localized spot that continues to indent the particle until phagocytosis is complete (orange arrows).

Scale bars, 10μm.

bioRxiv preprint doi: https://doi.org/10.64898/2026.04.30.721640; this version posted May 1, 2026. The copyright holder for this preprint (which was not certified by peer review) is the author/funder, who has granted bioRxiv a license to display the preprint in perpetuity. It is made available under aCC-BY 4.0 International license.

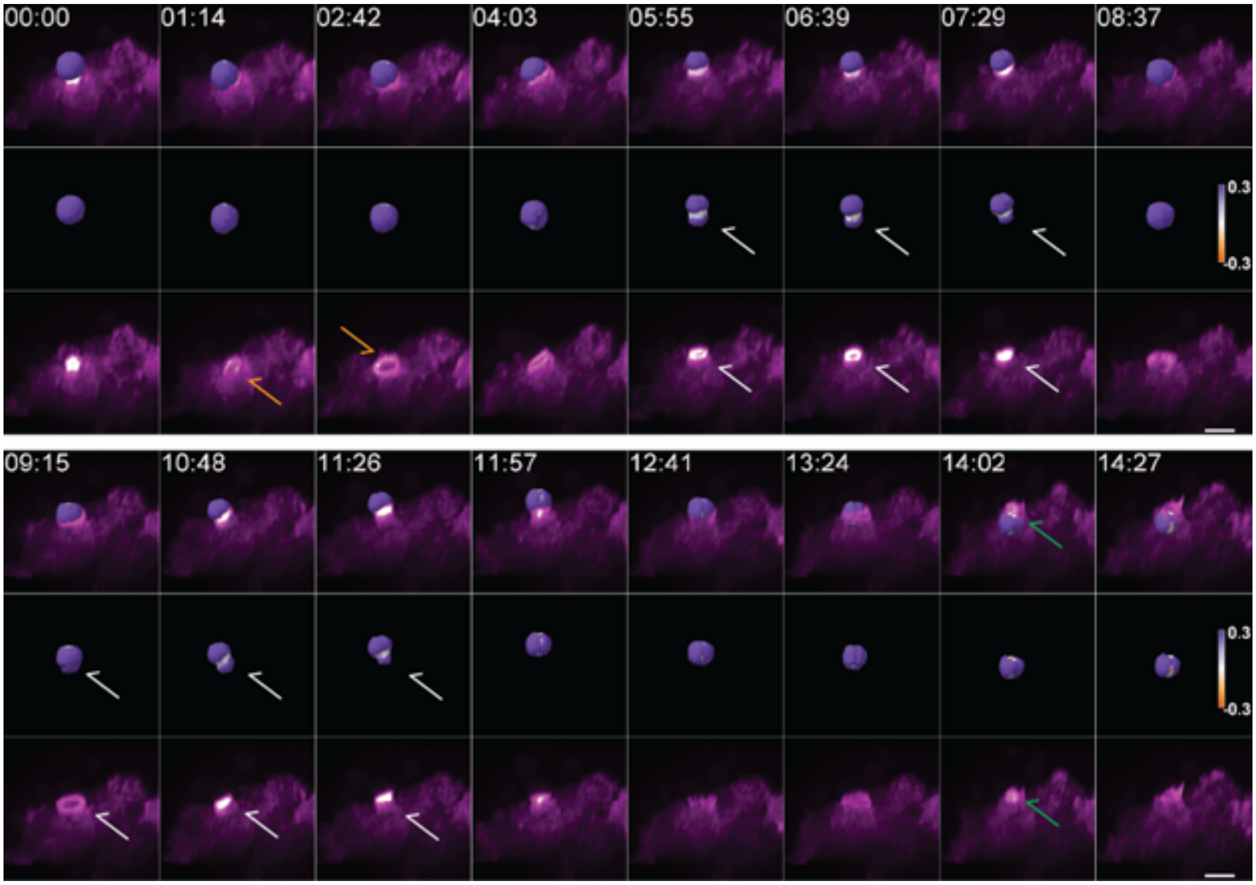

Supplemental Fig. 3 (related to Fig. 5): Myo1e/f dKO macrophage repeatedly pinches IgG-coated DAAMPs during failed FcγR-mediated phagocytic uptake.

Time series of a dKO RAW 264.7 macrophage expressing mEmerald–Lifeact, (magenta-white vMIP), attempting to eat an IgG-coated DAAMP rendered as an opaque isosurface with a diverging purple-orange colormap encoding local surface curvature (steradians). Early in the sequence, a localized rosette of F-actin–rich, podosome-like structures forms beneath the DAAMP. This superstructure subsequently disassembles, and a phagocytic cup containing podosome-like puncta becomes evident (orange arrows). Repeated failed uptake attempts are observed when podosome-like structures coalesce into a rosette-like configuration that constricts the DAAMP (white arrows) before sliding back and relaxing the constriction. In contrast, successful uptake is later observed during efficient progression and coalescence of F-actin-rich podosome-like structures above the upper hemisphere of the DAAMP (green arrows). Scale bar, 10μm. This time series was cropped to the region of interest and processed with surface dust filtering to display only the bead of interest.

bioRxiv preprint doi: https://doi.org/10.64898/2026.04.30.721640; this version posted May 1, 2026. The copyright holder for this preprint (which was not certified by peer review) is the author/funder, who has granted bioRxiv a license to display the preprint in perpetuity. It is made available under aCC-BY 4.0 International license.

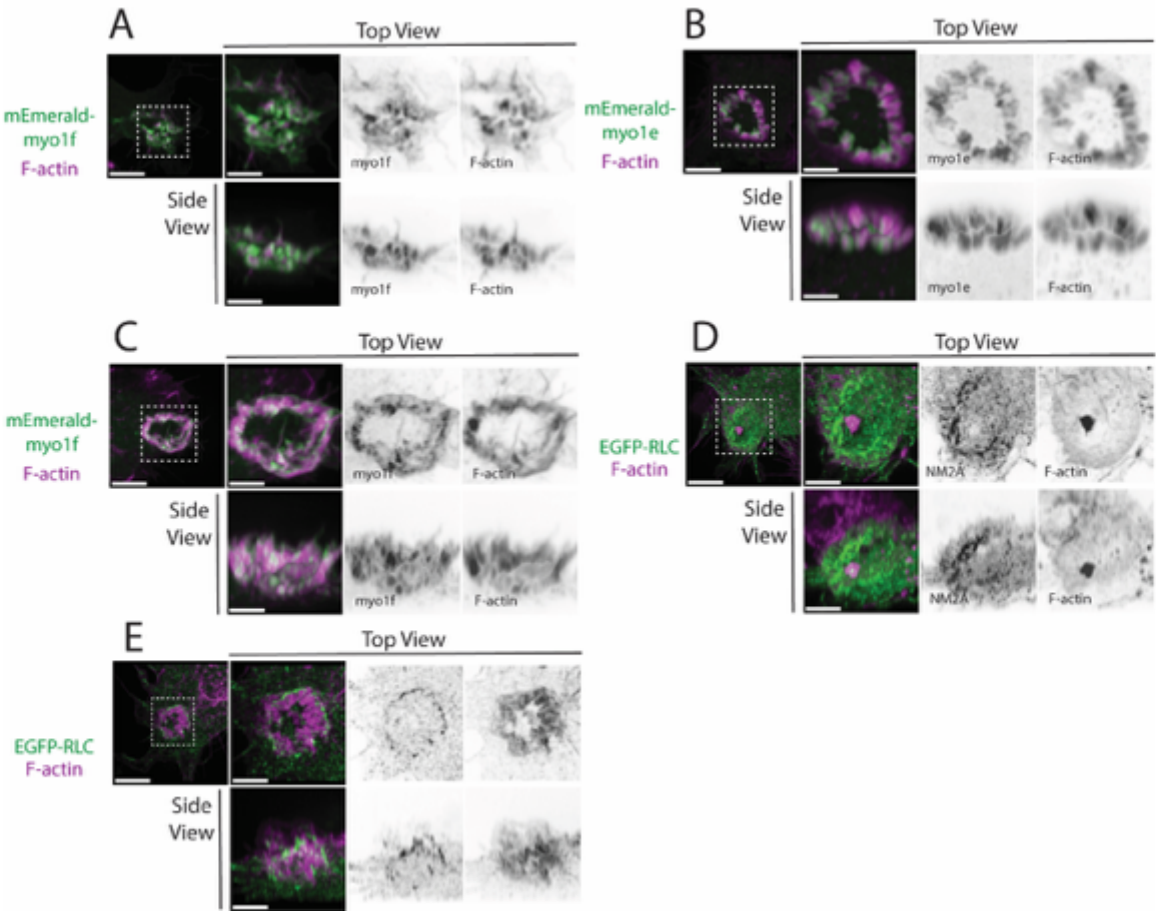

Supplemental Fig. 4:

(A) Myo1f localization to basal podosomes during early stages of cup formation. mEmerald-myo1f (green) localized to the tips of phalloidin-labeled F-actin hotspots (magenta) in basal podosomes.

(B-C) Myo1e and Myo1f localization to phagocytic actin teeth. mEmerald-Myo1e/f (green) colocalized to the tips of phalloidin-labeled F-actin (magenta) in actin teeth.

(D) NM2 localization relative to basal podosomes. EGFP-RLC labeled NM2 (green) did not colocalize with F-actin (magenta) in basal podosomes but was seen throughout the base of the cup.

(E) NM2 localization relative to phagocytic actin teeth. EGFP-RLC labeled NM2 (green) did not colocalize with F-actin (magenta) in actin teeth but was found in a condensed band at the base of the cup. All panels are RAW264.7 WT macrophages. Scale bars, 5µm, zoom scale bars, 2µm.

bioRxiv preprint doi: https://doi.org/10.64898/2026.04.30.721640; this version posted May 1, 2026. The copyright holder for this preprint (which was not certified by peer review) is the author/funder, who has granted bioRxiv a license to display the preprint in perpetuity. It is made available under aCC-BY 4.0 International license.

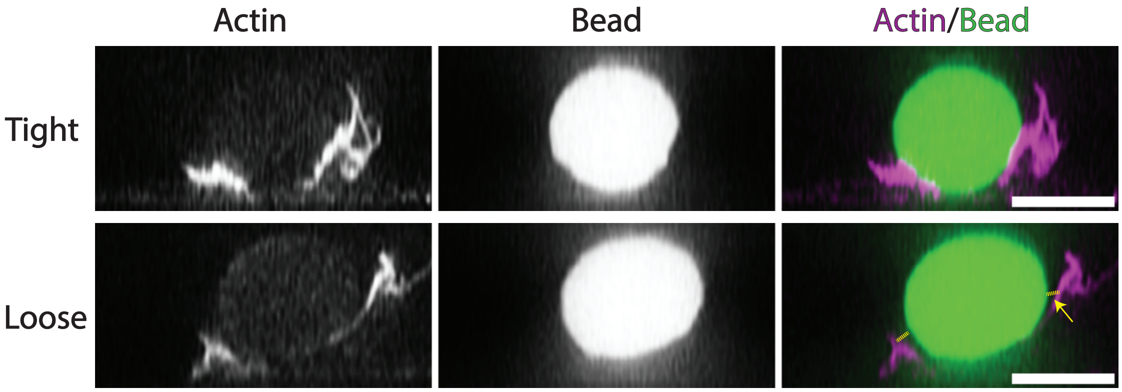

Supplemental Fig. 5 (related to Fig.6): Examples of tight or loose phagocytic cups quantified in Fig.6. An orthogonal (XZ) view of a phagocytic cup derived from a confocal Z-stack is shown in each montage. Actin is shown in grayscale (left panel) or magenta (right panel), DAAMP is shown in grayscale (middle panel) or green (right panel). Cells in which actin-rich phagocytic cups were in close contact with the bead were scored as “tight cups” while the cups that displayed widely open actin lamellipodia (yellow lines) and maintained a gap between the bead surface and cup rim (yellow arrow) were scored as “loose cups”. Scale bar, 10μm

bioRxiv preprint doi: https://doi.org/10.64898/2026.04.30.721640; this version posted May 1, 2026. The copyright holder for this preprint (which was not certified by peer review) is the author/funder, who has granted bioRxiv a license to display the preprint in perpetuity. It is made available under aCC-BY 4.0 International license.

Supplemental video legends

Supplemental Video 1 (related to Fig.3A). Composite video showing three panels of the same WT cell during engulfment of deformable beads: actin and beads (left), beads alone (middle), and actin alone (right).
Left panel shows a WT RAW 264.7 macrophage expressing mEmerald–Lifeact (blue isosurface) interacting with deformable IgG-coated DAAMPs, represented as an isosurface with a diverging purple–orange colormap representing local curvature measurements between -0.3 and 0.3 steradians. Middle panel shows beads only, right panel shows Lifeact only (magenta-white heatmap). Actin-rich podosome-like puncta form at the cell-bead interface and generate local target indentations. These structures subsequently reorganize into a ring-like rosette associated with pronounced constriction of the particle surface, followed by localized actin accumulation during final cup closure. Scale bar, 10 μm.

Supplemental Video 2 (related to Fig.5A). Composite video showing Myo1e/f dKO macrophage performing repeated constriction attempts during failed FcγR-mediated phagocytosis.
Left panel: a Myo1e/f dKO RAW 264.7 macrophage expressing mEmerald–Lifeact (F-actin; magenta-white heatmap) unsuccessfully attempting to engulf an IgG-coated DAAMP (purple-to-orange diverging colormap representing surface curvature). Middle panel: bead alone; right panel: actin alone. A localized actin-rich rosette forms beneath the particle and repeatedly constricts the target. Scale bar, 10 μm.

Supplemental Video 3 (related to Fig.S2). Composite video showing three panels of the same WT cell during engulfment of a deformable bead: actin and beads (left), beads alone (middle), and actin alone (right). Left panel: a WT RAW 264.7 macrophage expressing mEmerald–Lifeact (F-actin; magenta-white heatmap) unsuccessfully attempting to engulf an IgG-coated DAAMP (purple-to-orange diverging colormap representing surface curvature). Middle panel: bead alone; right panel: actin alone. Scale bar, 10 μm.

Supplemental Video 4 (related to Fig.S3). Composite video showing three panels of the same WT cell during engulfment of a deformable bead: actin and beads (left), beads alone (middle), and actin alone (right). A concentrated ring of actin forms around the bead causing repeated constriction of the bead; eventually the cell is able to successfully engulf the target. This sequence was cropped and surface dust filtered to display the bead of interest, and one frame was excluded due to another bead briefly coming into the field of view. Scale bar, 10 μm.
